# *BRAF* Inhibitors in *BRAF-Mutated* Colorectal Cancer: A Systematic Review

**DOI:** 10.3390/jcm13010113

**Published:** 2023-12-25

**Authors:** Wajeeha Aiman, Muhammad Ashar Ali, Samer Jumean, Ummul Asfeen, Jose Garcia, Murad Quirem, Amaar Ahmad, Mohammad Nabil Rayad, Osama Alkhlaifat, Bader Al Omour, Venkata S. Chemarthi, Michael Maroules, Gunwant Guron, Hamid Shaaban

**Affiliations:** 1 Department of Internal Medicine, Saint Michael’s Medical Center, New York Medical College, Newark, NJ 07102, USA; waiman@primehealthcare.com (W.A.); sjumean@primehealthcare.com (S.J.); uasfeen@primehealthcare.com (U.A.); jgarcia35@primehealthcare.com (J.G.); mqirem@primehealthcare.com (M.Q.); aahmad3@primehealthcare.com (A.A.); mrayad1@primehealthcare.com (M.N.R.); oalkhlaifat@primehealthcare.com (O.A.); 2 Department of Internal Medicine, St. Mary’s and St. Clare’s Hospitals, New York Medical College, Denville, NJ 07834, USA; 3 Department of Hematology/Oncology, Saint Michael’s Cancer Center, New York Medical College, Newark, NJ 07102, USAvchemarthi@primehealthcare.com (V.S.C.); gguron@primehealthcare.com (G.G.); hshaaban@primehealthcare.com (H.S.); 4 Department of Hematology/Oncology, Saint Mary’s Cancer Center, New York Medical College, Passaic, NJ 07055, USA; mmaroules@primehealthcare.com

**Keywords:** colorectal cancer, *BRAF* mutation, *BRAF* inhibitors, encorafenib, vemurafenib

## Abstract

Colorectal cancer (CRC) is the second-leading cause of cancer-related deaths globally. *BRAF* mutation is present in about 10% of CRC patients and is associated with a poor response to chemotherapy. These patients have a relatively poor prognosis. This review aims to assess the efficacy and safety of *BRAF* inhibitors in *BRAF*-*mutated* CRC patients. A literature search was performed on PubMed and Embase, and clinical trials relevant to *BRAF* inhibitors in CRC were included. Data were extracted for efficacy and safety variables. Two randomized clinical trials (*n* = 765) and eight non-randomized trials (*n* = 281) were included based on inclusion criteria. In RCTs, an overall response was reported in 23% of the patients treated with *BRAF* inhibitor-based regimens compared to 2.5% with control regimens. The hazard ratio of overall survival was also significantly better with triplet encorafenib therapy at 0.52 (95% CI = 0.39–0.70). In single-arm trials, ORR was 17% and 34% in two-drug and three-drug regimens, respectively. *BRAF* inhibitor-based regimens were safe and effective in the treatment of *BRAF*-mutated CRC. Large-scale randomized trials are needed to find a suitable population for each regimen. PROSPERO registration No. CRD42023471627.

## 1. Introduction

Colorectal cancer (CRC) is a universal public health issue. It is the third leading cause of cancer and the second most common cause of cancer-related deaths globally [1]. According to the statistics from the Global Cancer Observatory, colorectal cancer led to 9.4% of all deaths caused by cancer in 2020 and had an incidence rate of 10% compared to all other new cases of cancer in 2020 [2]. CRC, being a molecularly heterogeneous disease, has subtypes characterized by genetic alterations, with major efforts being made to reveal the molecular landscape of metastatic colorectal cancer (mCRC) [3]. CRC has a high prevalence of mutations in the mitogen-activated protein kinase pathway (MAPK pathway), including neuroblastoma *ras* viral oncogene homolog (*NRAS*) and Kirsten rat sarcoma 2 viral oncogene homolog (*KRAS*) sequence variations in roughly 50% of cancers and the v-raf murine sarcoma viral oncogene homolog B1 (*BRAF*) mutation, which is seen in 10%. *BRAF*, which is a biomarker in CRC, is a protein related to signal transduction for cell division and growth through the MAPKP. The *BRAF* proto-oncogene mutation leads to mutant CRC, a very distinct subtype of CRC with substitution in valine amino acid (V600E) due to transversion T1799A in exon 15, the most common mutation in *BRAF* [4]. *BRAF*^V600E^ mutation is more prevalent in right-sided colon cancer, females, the elderly, Caucasians, mucinous histology, mismatch repair defects, advanced-stage cancers, and those with a high tumor burden [4]. The *BRAF* mutation causes constitutive activation of the MAPKP, which leads to the high activating capacity of the MAPK pathway after a cascade of phosphorylation in the transcription of genes enhancing the differentiation, proliferation, inhibition of apoptosis, and survival of tumor cells [3].

**Figure 1:**
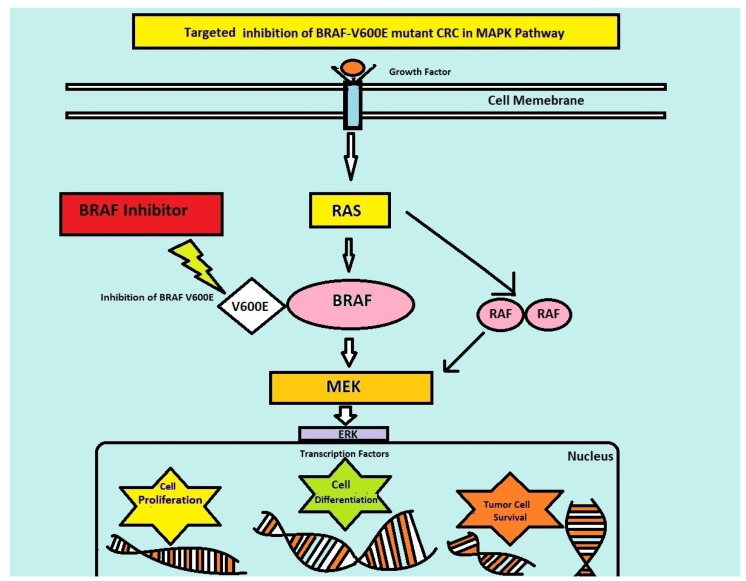
Targeted inhibition of *BRAF* pathway.

Standard chemotherapy and trials to intensify therapy have shown poor outcomes for *BRAF*^V600E^-mutated CRC. Even though the presence of *BRAF*^V600E^ mutations is a hallmark of a bad prognosis, it is a therapeutic target for treatment optimization and targeted therapy against *BRAF* mutation in *BRAF*-positive CRC. *BRAF* inhibitors have shown significant clinical activity against *BRAF*^V600E^-mutated tumor types like non-small cell lung cancer and melanoma. *BRAF* inhibition alone in *BRAF-*mutated CRC enhances feedback activation in other pathways, like *MAPKP* and epidermal growth factor receptor (*EGFR*) [5,6] (Figure 1).

Therefore, multiple target inhibitions including *EGFR* and *MEK* inhibitors are required to inhibit the proliferation of this type of cancer. Preclinical studies have shown the benefits of a combination of *BRAF* inhibitors with *MEK* inhibitors and anti-*EGFR* antibodies, which has led to clinical trials on these combinations.

This review aims to assess, acknowledge, and compile data from clinical trials regarding the safety and efficacy of *BRAF* inhibitor-based combinations in *BRAF-*mutated CRC for clinicians and to give directions for future clinical trials.

## 2. Materials and Methods

Cochrane Handbook [7] and PRISMA guidelines [8] were used to conduct this systematic review. The methodology for this systematic review was registered on PROSPERO with registration No. CRD42023471627.

### 2.1. Search Strategy

For this systematic review, we conducted a comprehensive literature search on PubMed (Medline) and Embase for clinical trials. We used keywords “colorectal cancer’’ and “B-raf protooncogene” from the inception of data to 15 August 2023. PICOS framework was used for the literature search [9] (Table S1).

### 2.2. Inclusion and Exclusion Criteria

We included all clinical trials conducted on adult patients (18+) with *BRAF-*mutated CRC treated with *BRAF* inhibitors. We included treatment regimens that showed clinical activity in *BRAF-*mutated CRC patients and were tolerated by most of the patients.

We excluded all preclinical studies, case reports, meta-analyses, review articles, and observational studies. We excluded clinical studies with more than 20% of patients with other types of cancer. We also excluded trials that could not be completed due to clinically ineffective regimens or high levels of toxicity.

### 2.3. Data Extraction

The primary efficacy outcomes of interest were overall response rate (ORR), median progression-free survival (mPFS), and median overall survival (mOS). Other secondary outcomes of interest were stable disease (SD), progressive disease (PD), complete response (CR), and partial response (PR). Safety data of interest were ≥ grade 3 adverse effects.

### 2.4. Risk of Bias Assessment

The risk of bias in selected clinical studies was assessed by two independent researchers using the Rob-2 tool for risk of bias assessment in RCTs [10].

## 3. Results

**Figure 2:**
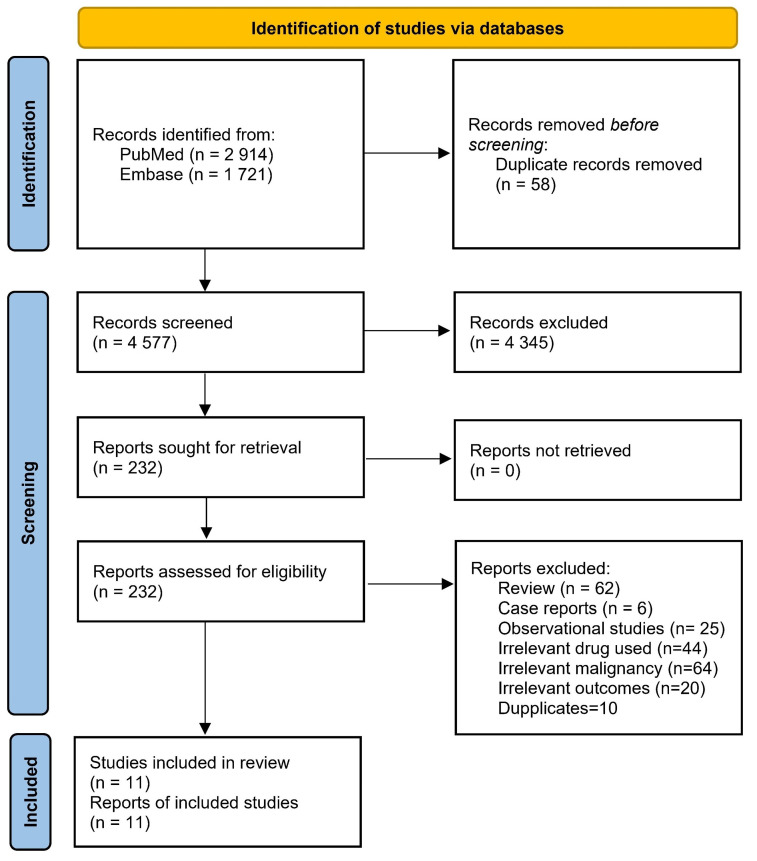
PRISMA flow chart of included studies.

A thorough literature search on PubMed and Embase found 4635 articles. After careful screening based on predefined inclusion and exclusion criteria, two randomized clinical trials (RCT, *n* = 765) [11,12], and nine non-randomized trials (nRCTs, *n* = 423) [3,13,14,15,16,17,18,19,20] were included, as in Figure 2.

### 3.1. Risk of Bias

**Figure 3:**
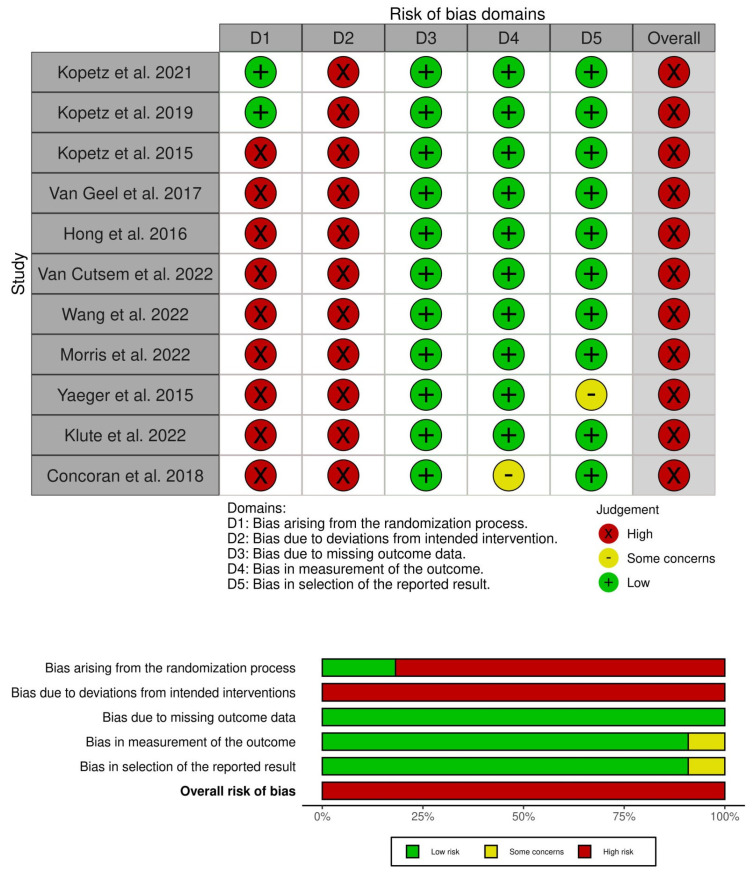
Risk of bias with ROB 2 tool [3,11,12,13,14,15,16,17,18,19,20].

The risk of bias was high in all studies included due to the lack of a placebo group and blinding. In two RCTs [11,12], the risk of bias was low for the randomization process, missing outcome data, measurement of the outcome, and selection of reported results. However, the risk of bias was high due to the open-label nature of these RCTs. The risk of bias was high in nRCTs [3,13,14,15,16,17,18,19,20], due to the lack of randomization and blinding, as shown in Figure 3.

### 3.2. BRAF Inhibitors in Relapsed/Refractory (R/R) mCRC

Ten clinical trials (*n* = 1093) were conducted on patients with R/R mCRC. A total of 21 patients were treated with *BRAF* inhibitors as monotherapy, 311 patients were treated with *BRAF* inhibitors in two-drug regimens, 447 patients were treated with *BRAF* inhibitors in three-drug regimens, and 302 patients were treated with chemotherapy regimens without *BRAF* inhibitors as a comparison group. Among the *BRAF*-treated patients, 619 patients were treated with encorafenib, 111 with dabrafenib, and 156 patients were treated with vemurafenib, as shown in Table 1.

#### 3.2.1. Efficacy

In two RCTs (*n* = 765) [11,12], CR, PR, and OR were 4/111 (3.6%), 25/111 (22.5%), and 37/161 (23%), respectively, in patients treated with a three-drug BRAF combination. CR, PR, and OR were 6/113 (5.3%), 17/113 (15%), and 23/113 (20.3%), respectively, in patients treated with two-drug BRAF regimens. CR, PR, and OR were 0/157 (0), 4/157 (2.5%), and 4/157 (2.5%), respectively, in patients treated with two-drug chemotherapy control regimens without BRAF inhibitors. In the RCT by Kopetz et al., 2021 [12], the hazard ratio (HR) for progression-free survival (PFS) was 0.5 (95% CI = 0.32–0.76) in favor of vemurafenib + cetuximab + irinotecan as compared to cetuximab + irinotecan. However, overall survival (OS) was similar among the two groups (HR = 0.77, 95% CI = 0.5–1.18). In the RCT by Kopetz et al., 2019 [11], HR for PFS was significantly better with triplet encorafenib therapy (encorafenib + binimetinib + cetuximab) 0.38 (95% CI = 0.29–0.49) as compared to the control group and 0.4 (95% CI = 0.31–0.52) as compared to doublet encorafenib therapy. HR for OS was also significantly better with triplet encorafenib therapy at 0.52 (95% CI = 0.39–0.70) as compared to control therapy, as shown in Table 2.

In nRCTs on two-drug regimens (*n* = 72) [18], CR, PR, and OR were 1/72 (1%), 11/72 (15%), and 12/72 (17%), respectively, on treatment with two-drug BRAF-based regimens. In nRCTs on three-drug regimens (*n* = 152), CR, PR, and OR were 3/152 (2%), 49/152 (32%), and 52/152 (34%), respectively, on treatment with three-drug regimens, as shown in Table 2.

#### 3.2.2. Safety

In two RCTs (*n* = 765) [11,12], ≥ grade 3 diarrhea was reported in 22/222 (10%), nausea in 10/222 (5%), vomiting in 9/222 (4%), abdominal pain in 13/222 (5.5%), anemia in 24/222 (11%), fatigue in 5/222 (2.2%), and rash in 1/222 (<1%) of patients treated with encorafenib + binimetinib + cetuximab. Adverse effects leading to discontinuation were reported in 7% of the patients. Higher than grade 3 diarrhea was reported in 4/216 (2%), nausea in 1/216 (0.5%), vomiting in 3/216 (1.4%), abdominal pain in 5/216 (2%), anemia in 9/216 (4%), fatigue in 9/216 (4%), and rash in 0/216 (0%) of patients treated with encorafenib + cetuximab. Treatment discontinuation due to adverse effects was reported in 8% of the patients. Higher than grade 3 diarrhea was reported in 19/193 (10%), nausea in 2/193 (1%), vomiting in 5/193 (3%), abdominal pain in 9/193 (5%), anemia in 8/193 (4%), fatigue in 8/193 (4%), and rash in 3/193 (2%) of patients treated with cetuximab and irinotecan OR cetuximab and FOLFIRI. Treatment discontinuation due to adverse effects in this arm was reported in 11% of the patients. In an RCT by Kopetz et al., 2021 [12], ≥grade 3 diarrhea was reported in 11/47 (23%), nausea in 9/47 (19%), vomiting in 5/47 (11%), abdominal pain in 2/47 (4%), anemia in 6/47 (13%), fatigue in 8/47 (17%), and rash in 1/47 (<1%) of patients treated with cetuximab + irinotecan. Treatment discontinuation due to adverse effects was reported in 8% of the patients. Higher than grade 3 diarrhea was reported in 6/46 (13%), nausea in 1/46 (2%), vomiting in 1/46 (2%), abdominal pain in 1/46 (2%), anemia in 0, fatigue in 7/46 (14%), and rash in 3/46 (6%) of patients treated with vemurafenib + cetuximab + irinotecan. Treatment discontinuation due to adverse effects was reported in 22% of the patients in this group. In other small-scale nRCTs, ≥ grade 3 diarrhea, rash, anemia, and treatment discontinuation due to toxicity were reported in 4%, 18%, 4%, and 7% of the patients, respectively, in patients treated with cobimetinib + vemurafenib. Higher than grade 3 diarrhea, rash, neutropenia, anemia, and adverse effects related to discontinuation were reported in 10%, 14%, 38%, 14%, and 0% of the patients, respectively, treated with vemurafenib + cetuximab + FOLFIRI, as shown in Table S2.

### 3.3. BRAF Inhibitor as First-Line Therapy in mCRC

In a phase II clinical trial (*n* = 95), all patients were treated with a three-drug regimen of encorafenib + cetuximab + binimetinib. CR, PR, and ORR were reported in 44/95 (46%), 41/95 (42%), and 3/95 (4%), respectively. The median PFS and OS were 5.8 (4.6–6.6) and 18.3 (14.1–21.1), respectively, as shown in Table 2. Severe side effects were reported in 49 (51.6%) of the patients. Higher than grade 3 diarrhea, nausea, vomiting, anemia, and intestinal obstruction were reported in 9 (9.5%), 8 (8.4%), 3 (3.2%), 10 (10.5%), and 14 (14.7%) patients, respectively, and 24% of the patients discontinued treatment due to adverse effects, as shown in Table S2.

### 3.4. Ongoing Clinical Trials

Randomized clinical trials are in progress for combinations of vemurafenib and irinotecan and cetuximab, and a combination of encurafenib and cetuximab. Other newer combination regimens of BRAF inhibitors include combinations of cetuximab, camrelizumab, HL-085, pembrolizumab, ZN-c3, hydroxychloroquine, binimetinib, and nivolumab, and non-randomized clinical trials are in progress, as shown in Table S3.

## 4. Discussion

The management of metastatic colon cancer with *BRAF* mutations includes utilizing aggressive treatment options like doublet and triplet chemotherapy regimens, as these patients have poor survival rates. Patients with *BRAF* mutations had a limited response to chemotherapy. Treatment strategies have evolved significantly in recent years; however, a clear standard of care has not been established. Ongoing research efforts have led to the emergence of *BRAF* inhibitors such as vemurafenib and encorafenib.

*BRAF* inhibitors initially showed significant survival benefits as monotherapy in *BRAF-*mutated solid tumors like melanoma [21]. However, the results were not repeated in the pilot study by Kopetz et al., 2015 [13], on the monotherapy of vemurafenib in *BRAF-*mutated colorectal cancer. Only minimal benefits were reported with the use of vemurafenib as a monotherapy. The likely reason was due to the over-activation of other pathways like *MEK*, *EGFR*, and phosphoinositide 3-kinase (*PI3K*). Similarly, EGFR inhibitors had activity in *BRAF-*mutated CRC patients; however, based on the results of a meta-analysis, they did not significantly improve outcomes in this population [22]. In a recent RCT by Stintzing et al., the EGFR inhibitor remains inferior to anti-VEGF (bevacizumab) in combination with chemotherapy in *BRAF-*mutated CRC patients [23]. Therefore, the addition of *BRAF* inhibitors was crucial to the treatment of *BRAF*-mutated CRC patients.

One of the earliest trials was conducted by Yaeger et al., 2015 [17], to study the efficacy and safety profile of vemurafenib and panitumumab (*EGFR* inhibitors) on patients who had previously progressed on at least one line of chemotherapy. Panitumumab was added to inhibit the reactivation of the *EGFR* signaling pathway. The combination showed clinical benefit in this patient population, but the magnitude was smaller, with a possible reason due to insufficient inhibition of other pathways like extracellular signal-regulated kinase (ERK) by the drug [24]. The paradoxical activation of other pathways including RAS and ERK remains a major challenge in BRAF inhibitors, which was addressed by combinations with other drugs. In this study, a safety assessment was conducted after six patients were enrolled due to concerns about potential overlapping toxicity between vemurafenib and panitumumab, particularly dermatologic toxicity. The results did not show any significant safety concerns and were well tolerated by most of the patients. Skin rashes generally correspond to the *EGFR*-targeted therapy efficacy, which does not apply to this case due to the opposing effects of these two agents in the skin used in this study. Liver enzymes were significantly elevated in 20% of the patients and should be followed up regularly in patients treated with this regimen.

Corcoran et al. initially tested a three-drug combination to inhibit *EGFR*, *MEK,* and *BRAF* pathways by combining dabrafenib, trametinib, and panitumumab [19]. The trial showed good response rates (21%) and an mPFS of 4.2 months. However, the benefit was limited due to safety concerns, especially serious dermatological toxicity. Van Geel et al. conducted a phase Ib trial on a three-drug regimen of encorafenib + cetuximab ± alpelisib. Dose-limiting toxicities were observed in three of the patients receiving dual therapy and two of the patients receiving triple therapy. Both the dual- and triple-combination therapies showed acceptable safety profiles. The clinical efficacy was better in the three-drug regimen as compared to the two-drug regimen. The trial showed that the combination of *BRAF* with *EGFR* and PI3Kα was a viable treatment option for this difficult-to-treat patient population. However, the study was non-randomized, the baseline characteristics of the patients were different among the two groups, and the ECOG score was higher in the two-drug regimen group. Therefore, the comparison between the two groups should be interpreted with caution.

Another combination of a *BRAF* inhibitor, an *EGFR* inhibitor, and chemotherapy was tested by Hong et al. combining vemurafenib, irinotecan, and cetuximab. At all dose levels of vemurafenib, anti-tumor activity was exhibited and mPFS was prolonged. Vemurafenib in combination with irinotecan and cetuximab was tolerated favorably, with only three patients experiencing dose-limiting toxicity. Fewer severe hepatotoxic events were reported in this three-drug regimen. The SWOG 1406 study conducted by Kopetz et al. expanded upon the same combination of vemurafenib, cetuximab, and irinotecan in the RCT. The trial demonstrated significant PFS improvement with the addition of a *BRAF* inhibitor to an *EGFR* inhibitor and a single-drug chemotherapy regimen. On subgroup analysis, the prior exposure to irinotecan and cetuximab did not significantly decrease the potential benefits of the above-mentioned regimen.

The BEACON trial was a large RCT that investigated the comparison of *BRAF* and *EGFR* inhibitors ± *MEK* inhibitor vs. the investigators’ choice of either cetuximab and FOLFIRI or cetuximab and irinotecan. The trial was not intended to compare the two experimental groups directly, but the overall survival analysis showed a hazard ratio for death that favored the triplet regimen (encorafenib + cetuximab + binimetinib). Adverse events were slightly higher in the three-drug regimen as compared to the two-drug regimen and were similar to side effects seen with previous trials on *BRAF*, *EGFR*, and *MEK* inhibitors. A real-world study by Boccaccino et al. returned the same response results for the three-drug regimen vs. the two-drug regimen as seen in the BEACON trial [25]. However, in the long-term updated results of the BEACON trial by Tabernero et al., response rates were slightly better with the three-drug regimen vs. two-drug regimen (27% vs. 20%), but there was no difference in median OS among the two groups (9.3 months vs. 9.3 months). The overall survival was significantly better than in the control group (9.3 vs. 5.9 months). Therefore, the two-drug regimen (encorafenib + cetuximab) can be used instead of the three-drug regimen or previous standard-of-care regimens.

Another dual combination of a *BRAF* inhibitor, vemurafenib, and a *MEK* inhibitor, cobimetinib, was not tested in the SWOG or BEACON trials. This combination showed anti-tumor activity in pretreated patients with no new safety concerns. However, this was a small-scale study, and the combination needs to be tested in RCTs to compare with the *BRAF* and *EGFR* inhibitor combination.

After promising results of the *BRAF* + *EGFR* inhibitor combination in the BEACON trial, the IMPROVEMENT trial combined these drugs with FOLFIRI [3]. A total of 85% of patients achieved an objective response with the combination therapy, while 100% achieved disease control that was significantly better than prior tolerated regimens. The trial also included patients with ECOG 2, who were underrepresented in prior studies. The regimen was well tolerated by most of the patients and required dose reductions in 9/21 patients.

ANCHOR CRC assessed the triple combination of encorafenib, binimetinib, and cetuximab in a first-line setting for the first time without chemotherapy [20]. The results were similar to chemotherapy + bevacizumab in the FIRE-4.5 study [23]. This trial showed a feasible chemotherapy-free option for first-line therapy in *BRAF-*mutated patients. However, more large-scale studies are needed to find a subgroup of patients better suited for chemotherapy-free regimens and compare it with chemotherapy-based regimens.

The BRAF mutation is generally considered a poor prognostic factor; however, different subsets of BRAF-mutated patients might have different prognoses and responses to treatment. Therefore, the development of biomarkers to predict response and prognosis is important, as is identifying patients who might benefit from the intensification of therapy. In a retrospective study by Loupakis et al., a scoring system “BeCool” was devised for *BRAF-*mutated patients to predict prognosis. The scoring was majorly based on CA19-9, lactate dehydrogenase levels, ECOG performance, neutrophil to lymphocyte ratio, metastatic sites, and grading of cancer, as they were independently associated with significant changes in OS. Patients with a low score (0–4) had an mOS of 29.6, intermediate (5–8) had 15.5, and high (9–16) had an mOS of 6.6 months. However, most of the patients in this study were not treated with BRAF inhibitors [26]. An exploratory study by Elez et al. suggested that the *RNF43* mutation in *BRAF-*mutated patients is a favorable predictor of response to *BRAF* inhibitors in *BRAF-*mutated microsatellite stable (MSS) CRC patients. The ORR was 73% in *RNF43*-mutated vs. 31% in *RNF43*-wild-type CRC patients treated with *BRAF* inhibitors [27]. A prospective study by Ros et al. found that a high plasmatic allele fraction (AF) of *BRAF* was associated with worse PFS and OS as compared to low AF in patients with *BRAF*-mutated CRC treated with *BRAF* and anti-*EGFR* inhibitors. Thus, patients with high AF might benefit from the intensification of *BRAF* inhibitor therapy [28]. In a retrospective study by Kopetz et al., *BRAF-*mutated CRC patients with the consensus molecular subtype (CMS-4) and *BRAF* mutant (BM1) subtypes had better responses to triplet therapy as compared to doublet therapy. Thus, this highlighted the potential of using CMS and BM classification as predictors of response to different regimens [29].

Microsatellite instability (MSI) and *BRAF* gene mutations are closely related to each other and coexist in about 52% of the patients with a *BRAF* mutation [30]. Checkpoint inhibitors like nivolumab, ipilimumab, and pembrolizumab have shown promising results in advanced MSI colorectal cancers. A combination of checkpoint inhibitors with *BRAF* inhibitors is also a feasible combination to test in *BRAF-*mutated patients. In clinical studies, patients with microsatellite stability (MSS) treated with encorafenib + cetuximab had transient microsatellite instability (MSI) [4]; therefore, Morris et al. conducted a phase I/II study (*n* = 26) on the addition of nivolumab to encorafenib + cetuximab in MSS patients. Based on interim results presented in the ASCO annual meeting of 2022, the combination was well tolerated and was clinically active. Large-scale RCTs are needed to assess the significance of nivolumab or other PD-1/PD-L1 inhibitors in these patients. There is another phase III clinical trial, NCT05217446, in progress to assess the efficacy and safety of *BRAF* inhibitors ± pembrolizumab in *BRAF* and MSI-H colorectal cancer patients.

## 5. Conclusions

*BRAF* inhibitors, encorafenib, and vemurafenib, were well tolerated by most of the patients in combination with *EGFR* inhibitors, *MEK* inhibitors, and FOLFIRI drugs. In RCTs, *BRAF-*based three-drug and two-drug regimens were more effective than prior standard-of-care chemotherapy regimens in R/R patients with *BRAF-*mutated CRC. In the long-term results, survival rates of *BRAF-*based two-drug regimens (*BRAF* + *EGFR*) were similar to those of *BRAF-*based three-drug regimens with better safety profiles. Therefore, two drug regimens can be used as a standard of care in *BRAF-*mutated R/R patients. Among nRCTs, a combination of *BRAF* with *EGFR* and FOLFIRI drugs showed the highest response and survival rates in R/R *BRAF-*mutated CRC patients. The *BRAF-*based, three-drug, chemotherapy-free regimen also showed clinical activity with acceptable toxicity as a first-line therapy. Large-scale RCTs are needed to compare *BRAF*-based regimens and find appropriate populations for *BRAF*-based regimens.

## 6. Limitation

There were no double-blinded large-scale clinical studies available to include in this systematic review; therefore, the risk of bias was high in the included studies. Most of the *BRAF-*based regimens, including *BRAF* combinations with chemotherapy and checkpoint inhibitors, were only tested in small-scale, single-arm studies, and randomized studies are in progress. Since most of the treatment regimens were only tested in small-scale, single-arm studies, a meaningful meta-analysis could not be conducted. Despite these limitations, the article was able to provide a comprehensive review of the current evidence available on *BRAF* inhibitors in *BRAF*-mutated CRC.

## Figures and Tables

**Table 1:** Baseline characteristics of patients included in clinical trials on *BRAF* inhibitors in colorectal cancer.

Author et al.	NCT	Phase of Trial	Treatment Regimen	Participants (*n*)	Age Median (Years)	Sex		ECOG Performance	Location of Primary Tumor				Involvement of ≥3 Organs (and/or Locations Involved)	Number of Previous Lines of Therapies	High MSI
						Male	Female		Left Side of Colon (or Rectum)	Right Side of Colon (or Appendix)	Colon	Rectal			
Randomized clinical trials on relapsed/refractory patients															
Kopetz et al., 2019 [11]	NCT02928224	Phase III	Encorafenib + Binimetinib + Cetuximab	224	62	105	119	0 = 116 1 = 108	79	126	19	NA	Involvement of ≥3 organs = 110, Liver = 144	1 = 146 2 = 78	22
			Encorafenib + Cetuximab	220	61	115	105	0 = 112 1 = 104	83	110	27	NA	Involvement of ≥3 organs = 103, Liver = 134	1 = 123 2 = 97	19
			Cetuximab and Irinotecan OR Cetuximab and FOLFIRI (Control)	221	60	94	127	0 = 108 1 = 113	68	119	34	NA	Involvement of ≥3 organs = 98, Liver = 128	1 = 145 2 = 76	12
Kopetz et al., 2021 [12]	NCT02164916	Phase II	Cetuximab + Irinotecan	50	61.9	13	37	0 = 23 1 = 27	17	30	NA	3	Distant Lymph Nodes = 14, Lung = 21, Liver = 28, Bone = 2, Peritoneum = 16	1 = 25 2 = 17	7
			Vemurafenib + Cetuximab + Irinotecan	50	59.7	29	21	0 = 24 1 = 26	8	36	NA	6	Distant Lymph Nodes = 13, Lung = 21, Liver = 33, Bone = 2, Peritoneum = 16	1 = 27 2 = 20	6
Non-randomized clinical trials on relapsed/refractory patients															
Hong et al., 2016 [15]	NCT01787500	Phase IB	Cetuximab and Irinotecan with escalating doses of Vemurafenib	19	63	10	9	0 = 4 1 = 14	NA	1	18	NA	NA	Median = 2, 14 = Irinotecan, 8 = Cetuximab, 1 = *BRAF*	3
Klute et al., 2022 [18]	NA	Phase II	Cobimetinib + Vemurafenib	30	62	11	19	0 = 12 1 = 13	10	15	NA	NA	NA	Radiation = 7 Systemic = 7	1
Wang et al., 2022 [3]	NCT03727763	Phase II	Vemurafenib + Cetuximab + FOLFIRI	21	49	13	8	0 = 4 1 = 7	4	11	NA	6	Liver = 5, Lung = 5, Distant Lymph Nodes = 8, Abdominopelvic Cavity = 11, Other = 4	1 = 12, 2 = 8, 3 = 1	NA
Yaeger et al., 2015 [17]	NA	Pilot Trial	Vemurafenib + Panitumumab	15	62	7	8	0 = 4 1 = 11	4	10	NA	NA	NA	1 = 7, 2 = 8	NA
Kopetz et al., 2015 [13]	NCT00405587	Phase II Pilot	Vemurafenib as Single Agent	21	65	11	10	0 = 11 1 = 10	NA	NA	20	1	NA	0 = 1, 1 = 1 2+ = 17	NA
Corcoran et al., 2018 [19]	NCT01750918	Phase I	Dabrafenib + Panitumomab	20	58	9	11	0 = 13 1 = 7	4	14	18	2	NA	0 = 4, 1 = 8, 2+ = 8	NA
			Trametinib + Panitumomab	31	57	13	18	0 = 17 1 = 14	10	16	26	5	NA	0 = 1, 1 = 14, 2+ = 16	NA
			Dabrafenib + Trametinib + Panitumomab	91	60	33	58	0 = 47 1 = 44	19	57	76	15	NA	0 = 21, 1 = 27, 2+ = 43	11
Morris et al., 2022 [16]	NCT04017650	Phase I/II	Encorafenib, Cetuximab, and Nivolumab	26	59	12	14	NA	NA	NA	NA	NA	NA	NA	NA
Van Geel et al., 2017 [14]	NCT01719380	Phase IB	Encorafenib + Cetuximab	26	63	11	15	0 = 8 1 = 16	NA	NA	24	2	Liver = 15, Peritoneum = 5	1 = 7, 2+ = 19	NA
			Encorafenib + Cetuximab + Alpelisib	28	59	10	18	0 = 18 1 = 10	NA	NA	25	3	Liver = 16, Peritoneum = 8	1 = 10, 2+ = 18	NA
Non-randomized clinical trial on newly diagnosed patients															
Van Cutsem et al., 2023 [20]	NCT03693170	Phase II	Encorafenib + Binimetinib + Cetuximab	95	65	44	51	0 = 43, 1 = 52	37	57	NA	NA	Peritoneal = 46, Liver = 52, Lung = 35	19% Received Prior Adjuvant Systemic Treatment	NA

**Table 2:** Efficacy of BRAF inhibitor-based regimens in BRAF-mutated CRC.

Author	Regimen	Median OS	Median PFS	ORR	CR	PR	PD	SD
Randomized clinical trials on relapsed/refractory patients								
S. Kopetz et al., 2019 [11]	Encorafenib + Binimetinib + Cetuximab	9.0 months	4.3 months	29/111 (26%)	4/111 (4%)	25/111 (23%)	11/111 (10%)	47/111 (42%)
	Encorafenib + Cetuximab	8.4 months	4.2 months	23/113 (20%)	6/113 (5%)	17/113 (15%)	8/113 (7%)	61/113 (54%)
	Cetuximab and Irinotecan OR Cetuximab and FOLFIRI	5.4 months	1.5 months	2/107 (2%)	0/107	2/107 (2%)	36/107 (34%)	31/107 (29%)
Kopetz et al., 2021 [12]	Cetuximab + Irinotecan	5.9 months	2 months	2 (4%)	NA	NA	38/50 (76%)	NA
	Vemurafenib + Cetuximab + Irinotecan	9.6 months	4.2 months	8 (17%)	NA	NA	31/50 (62%)	NA
Non-randomized clinical trials on relapsed/refractory patients								
Hong D.S., et al., 2016 [15]	Cetuximab and Irinotecan with escalating doses of Vemurafenib	NA	7.7 months	6/17 (35%)	0	6/17 (35%)	2/17 (12%)	9/17 (53%)
Klute K. A. et al., 2022 [18]	Cobimetinib + Vemurafenib	8.95 months	3.6 months	8/27 (30%)	0	8/27 (30%)	NA	6/27 (22%)
Wang, Z. et al., 2022 [3]	Vemurafenib + Cetuximab + FOLFIRI	15.4 months	9.7 months	17/21 (81%)	2/21 (9.5%)	15/21 (71%)	10/21 (47%)	3/21 (14%)
Yaeger, R. et al., 2015 [17]	Vemurafenib + Panitumumab	7.6 months	3.2 months	2/15 (13%)	0	2/15 (13%)	2/15 (13%)	2/15 (13%)
Kopetz et al., 2015 [13]	Vemurafenib as Single Agent	7.7 months	2.1 months	1/21 (5%)	0	1/21 (5%)	NA	7/21 (33%)
Corcoran et al., 2018 [19]	Dabrafenib + Panitumomab	13.2 months	3.5 months	2/31 (10%)	1/31 (5%)	1/31 (5%)	2/31 (10%)	16 (80%)
	Trametinib + Panitumomab	8.2 months	2.6 months	0	0	0	12/31 (39%)	17/31 (55%)
	Dabrafenib + Trametinib + Panitumomab	9.1 months	4.2 months	19/91 (21%)	1/91 (1%)	18/91 (20%)	8/91 (9%)	59/91 (65%)
Morris et al., 2022 [16]	Encorafenib, Cetuximab, and Nivolumab	11.4 months	7.3 months	12/26 (45%)	NA	NA	NA	NA
Van Geel R. et al., 2017 [14]	Encorafenib + Cetuximab	NA	3.7 months	5/26 (19%)	1/26 (4%)	4/26 (15.5%)	4/26 (15.5%)	15/26 (58%)
	Encorafenib + Cetuximab + Alpelisib	NA	4.2 months	5/28 (18%)	0	5/28 (18%)	1/28 (3.6%)	21/28 (75%)
Non-randomized clinical trial on newly diagnosed patients								
Van Cutsem et al., 2023 [20]	Encorafenib + Binimetinib + Cetuximab	18.3 months	5.8 months	45/95 (47%)	0	45/95 (47%)	48/95 (50%)	37/95 (41%)

CR = complete response, PR = partial response, ORR = overall response, OS = overall survival, PFS = progression-free survival.

## Data Availability

No new data were created or analyzed in this study. Data sharing is not applicable to this article.

## Supplementary Materials

The following supporting information can be downloaded at: https://www.mdpi.com/article/10.3390/jcm13010113/s1, Table S1: PICOS table and database search.; Table S2: Safety of *BRAF* inhibitor-based regimens in CRC patients; Table S3. Ongoing clinical trials on *BRAF* inhibitor-based regimens in CRC.
