# Supplementary material for: BRAF Inhibitors in BRAF-Mutated Colorectal Cancer: A Systematic Review
Source: J Clin Med. 2023 Dec 25;13(1):113. doi: 10.3390/jcm13010113 (PMC10779564; doi:10.3390/jcm13010113)
Supplement: Supplementary file 1 [file jcm-13-00113-s001.zip › jcm-2678376-supplementary.pdf]

Table S1. PICOS table and data base search.

<table>
<tr><td>P</td><td>I</td><td>C</td><td>O</td><td>S</td></tr>
<tr><td>"Colorectal Neoplasms"[Mesh]</td><td>"Proto-Oncogene Proteins B-raf"[Mesh]</td><td></td><td></td><td></td></tr>
<tr><td>Colorectal Neoplasm

Neoplasms, Colorectal

Colorectal Tumors

Colorectal Tumor

Colorectal Cancer

Colorectal Cancers

Colorectal Carcinoma

Colorectal Carcinomas</td><td>Proteins B-raf, Proto-Oncogene

Proto Oncogene Proteins B raf

BRAF Kinase

BRAF Kinases

B-raf Kinases

B raf Kinases

B-raf Kinase

B raf Kinase

Proto-Oncogene Protein B-raf

Protein B-raf, Proto-Oncogene

Proto Oncogene Protein B raf</td><td></td><td></td><td></td></tr>
<tr><td colspan="5">PubMed:
((((((((((("Proto-Oncogene Proteins B-raf"[Mesh]) OR (Proteins B-raf, Proto-Oncogene)) OR (Proto Oncogene Proteins B raf)) OR (BRAF Kinase)) OR (BRAF Kinases)) OR (B-raf Kinases)) OR (B raf Kinases)) OR (B-raf Kinase)) OR (B raf Kinase)) OR (Proto-Oncogene Protein B-raf)) OR (Protein B-raf, Proto-Oncogene)) OR (Proto Oncogene Protein B raf)) AND ((((((((("Colorectal Neoplasms"[Mesh]) OR (Colorectal Neoplasm)) OR (Neoplasms, Colorectal)) OR (Colorectal Tumors)) OR (Colorectal Tumor)) OR (Colorectal Cancer)) OR (Colorectal Cancers)) OR (Colorectal Carcinoma)) OR (Colorectal Carcinomas))
2,914</td></tr>
<tr><td colspan="5">Embase:</td></tr>
</table>

('colorectal cancer'/exp/mj OR 'cancer of colon and rectum' OR 'cancer of rectum and colon' OR 'cancer of the colon and rectum' OR 'cancer of the colon and the rectum' OR 'cancer of the rectum and colon' OR 'cancer of the rectum and the colon' OR 'colo-rectal cancer' OR 'colo-rectal carcinogenesis' OR 'colo-rectal malignancies' OR 'colo-rectal malignancy' OR 'colorectal cancer' OR 'colorectal cancerogenesis' OR 'colorectal carcinogenesis' OR 'colorectal malignancies' OR 'colorectal malignancy' OR 'malignancies of the colon and rectum' OR 'malignancy of colon and rectum' OR 'malignancy of the colon and rectum' OR 'recto-colonic cancer' OR 'rectocolonic cancer') AND ('b raf kinase inhibitor'/exp/mj OR 'b raf kinase inhibitor' OR 'braf kinase inhibitor' OR 'b raf kinase'/exp/mj OR 'b raf kinase' OR 'b raf protein' OR 'braf protein' OR 'kinase braf' OR 'protein b raf' OR 'protein braf' OR 'proto oncogene proteins b raf' OR 'proto-oncogene proteins b-raf') AND ('clinical trial'/exp/mj OR 'clinical drug trial' OR 'clinical trial' OR 'major clinical trial' OR 'trial, clinical')
1,721

Table S2: Safety of BRAF inhibitors in CRC:

Author	Regimen	Adverse effects grade >/= 3																
		Diarrhea	Nausea	Vomiting	Abdominal Pain	Decreased Appetite	Constipation	Asthenia	Pyrexia	Rash	Arthralgia	Myalgia	ALT	AST	Cr	Anemia	Neutropenia	Fatigue
**Randomized clinical trials on relapsed/refractory patients**																		
Kopetz et. al. 2019	Encorafenib + Binimetinib + Cetuximab	22/222 (10%)	10/222 (5%)	9/222 (4%)	13/222 (6%)	4/222 (2)	0	7/222 (3%)	4/222 (2%)	1/222 (<1%)	0	0	4/222 (2%)	4/222 (2%)	10/222 (5%)	24/222 (11%)	NA	5/222 (2%)
	Encorafenib + Cetuximab	4/216 (2%)	1/216 (<1%)	3/216 (1%)	5/216 (2%)	3/216 (1%)	0	7/216 (3%)	2/216 (1%)	0	2/216 (1%)	1/216 (<1%)	0	3/216 (1%)	5/216 (2%)	9/216 (4%)	NA	9/216 (4%)
	Cetuximab and Irinotecan OR Cetuximab and FOLFIRI (Control)	19/193 (10%)	2/193 (1%)	5/193 (3%)	9/193 (5%)	6/193 (3%)	2/193 (1%)	9/193 (5%)	1/193 (1%)	3/193 (2%)	0	0	5/193 (3%)	3/193 (2%)	2/193 (1%)	8/193 (4%)	NA	8/193 (4%)
Kopetz et al 2021	Cetuximab + Irinotecan	11/47 (23%)	9/47 (19%)	5/47 (11%)	2/47 (4%)	NA	NA	NA	NA	1/47 (2%)	3/47 (6%)	2/47 (4%)	NA	NA	NA	6/47 (13%)	14/47 (30%)	8/47 (17%)
	Vemurafenib + Cetuximab + Irinotecan	6/46 (13%)	1/46 (2%)	1/46 (2%)	1/46 (2%)	NA	NA	NA	NA	3/46 (6%)	0	0	NA	NA	NA	0	NA	7/46 (14%)
**Non-randomized clinical trials on relapsed/refractory patients**																		
Hong et al. 2016	Cetuximab + Irinotecan + Vemurafenib	5/17 (30%)	1/17 (6%)	0	NA	NA	NA	NA	NA	1/17 (6%)	2/17 (12%)	NA	NA	NA	NA	2/17 (12%)	3/17(17%)	2/17 (12%)
Klute et al. 2022	Cobimetinib + Vemurafenib	1/27(4%)	NA	NA	NA	NA	NA	NA	NA	5/27 (18%)	NA	NA	1/27 (4%)	1/27 (4%)	NA	1/27 (4%)	NA	1/27 (4%)
Wang et al. 2022	Vemurafenib + Cetuximab + FOLFIRI	2/21 (10%)	(0/21)	(0/21)	NA	NA	NA	NA	0/21	3/21 (14%)	1/21 (5%)	1/21 (5%)	1/21 (5%)	2/21 (10%)	NA	3/21 (14%)	8/21 (38%)	2/21 (9%)
Yaeger et al. 2015	Vemurafenib + Panitumumab	0	0/15	NA	NA	NA	NA	NA	NA	0	0	NA	3/15 (20%)	3/15 (20%)	NA	NA	1/15 (7%)	1/15 (7%)
van Geel et al. 2017	Encorafenib + Cetuximab	NA	0	2/26 (7.7%)	3/26 (11.5%)	0	1/26 (3.8%)	NA	0	0	NA	0	NA	NA	NA	NA	NA	3/26 (11.5%)
	Encorafenib + Cetuximab + Alpelisib	NA	1/28 (3.6%)	0	1/28 (3.6%)	1/28 (3.6%)	0	NA	1/28 (3.6%)	0	NA	0	NA	NA	NA	NA	NA	1/28 (3.6%)
Morris, et al. 2022	Encorafenib, Cetuximab, and Nivolumab	NA	NA	NA	NA	NA	NA	NA	NA	4%	NA	NA	NA	NA	NA	NA	NA	NA
S. Kopetz et. al.2015	Vemurafenib as Single Agent	1/15 (6%)	0/15	1/15 (6%)	NA	NA	NA	NA	0/15	2/15 (12%)	1/15 (6%)	NA	NA	NA	NA	NA	NA	1/15 (6%)
**Non-randomized clinical trial on newly diagnosed patients**																		
Van Cutsem et al. 2022	Encorafenib + Binimetinib + Cetuximab	9/95 (9.5%)	8/95 (8.4%)	3/95 (2%)	4/95 (4.2%)	3/95 (2%)	0	2/95 (2%)	1 (1.1%)	1 (1.1%)	NA	NA	NA	NA	NA	10/95 (10%)	NA	0

Table S3: Ongoing clinical trials on BRAF inhibitors in CRC.

NCT	N	Phase	Type of study	Population	Intervention	Outcome	Status
NCT05019534	12	I	Single arm	BRAF V600E-mutant /MSS type mCRC.	Vemurafenib, Cetuximab and Camrelizumab (VCC)	Efficacy and Safety	Recruiting
NCT05540951	78	III	Randomized	unresectable BRAF V600E-mutated mCRC	VIC Regimen (Vemurafenib/Irinotecan/Cetuximab) vs Bevacizumab + Chemotherapy	Efficacy and Safety	Recruiting
NCT05233332	186	II	Non-Randomized Sequential Assignment	mCRC	HL-085 with Vemurafenib	Efficacy and Safety	Not yet recruiting
NCT05217446	104	II	Parallel Assignment	BRAF V600E-mutant MSI-H/dMMR mCRC	Encorafenib, cetuximab + pembrolizumab versus pembrolizumab	Efficacy and Safety	Recruiting
NCT05743036	82	I	Randomized Sequential Assignment	mCRC	Encorafenib +Cetuximab + ZN-c3	Efficacy and Safety	Not yet recruiting
NCT05004350	103	II	Randomized, Parallel Assignment	BRAF V600E	Encorafenib + Cetuximab Vs Irinotecan + Cetuximab or (FOLFIRI) + Cetuximab	Efficacy and Safety	Recruiting
NCT05576896	43	II	Single Group Assignment	BRAF-mutated mCRC Refractory	Hydroxychloroquine + Encorafenib + Cetuximab or Panitumumab	Efficacy and Safety	Recruiting
NCT05510895	48	II	Single Group Assignment	BRAF V600E Mutated/pMMR Localized CRC	Encorafenib, Binimetinib and Cetuximab	Efficacy and Safety	Not yet recruiting
NCT04017650	38	II	Single Group Assignment	MSS BRAFV600E mCRC	Encorafenib, Cetuximab, and Nivolumab	Efficacy and Safety	Recruiting
NCT04607421	765	III	Randomized, Parallel Assignment	BRAF V600E-mutant mCRC	Encorafenib + Cetuximab with/without chemotherapy vs SOC	Efficacy and Safety	Recruiting
NCT05308446	84	II	Randomized, Parallel Assignment	Unresectable or mCRC BRAF mutated	Encorafenib, Cetuximab with/without Nivolumab	Efficacy and Safety	Recruiting
NCT05706779	30	II	Single Group Assignment	BRAF V600E-mutant Localized Colon or Upper Rectum Cancer	Encorafenib + Cetuximab	Efficacy	Not yet recruiting

MSS: microsatellite stability, MSI-H: microsatellite instability-high, dMMR: deficient mismatch repair, pMMR: Proficient mismatch repair, FOLFIRI: 5-fluorouracil (5-FU)/Folinic Acid (FA)/Irinotecan
